# Social Media in Professional Medicine: New Resident Perceptions and Practices

**DOI:** 10.2196/jmir.5612

**Published:** 2016-06-09

**Authors:** Cedric Lefebvre, Jason Mesner, Jason Stopyra, James O'Neill, Iltifat Husain, Carol Geer, Karen Gerancher, Hal Atkinson, Erin Harper, William Huang, David M Cline

**Affiliations:** ^1^ Wake Forest School of Medicine Department of Emergency Medicine Winston Salem, NC United States; ^2^ Wake Forest School of Medicine Department of Radiology Winston Salem, NC United States; ^3^ Wake Forest School of Medicine Department of Obstetrics and Gynecology Winston Salem, NC United States; ^4^ Wake Forest School of Medicine Department of Internal Medicine Winston Salem, NC United States; ^5^ Wake Forest School of Medicine Department of Dermatology Winston Salem, NC United States

**Keywords:** social media, professionalism, physicians, education

## Abstract

**Background:**

For younger generations, unconstrained online social activity is the norm. Little data are available about perceptions among young medical practitioners who enter the professional clinical arena, while the impact of existing social media policy on these perceptions is unclear.

**Objective:**

The objective of this study was to investigate the existing perceptions about social media and professionalism among new physicians entering in professional clinical practice; and to determine the effects of formal social media instruction and policy on young professionals’ ability to navigate case-based scenarios about online behavior in the context of professional medicine.

**Methods:**

This was a prospective observational study involving the new resident physicians at a large academic medical center. Medical residents from 9 specialties were invited to participate and answer an anonymous questionnaire about social media in clinical medicine. Data were analyzed using SAS 9.4 (Cary, NC), chi-square or Fisher’s exact test was used as appropriate, and the correct responses were compared between different groups using the Kruskal–Wallis analysis of variance.

**Results:**

Familiarity with current institutional policy was associated with an average of 2.2 more correct responses (*P*=.01). Instruction on social media use during medical school was related to correct responses for 2 additional questions (*P*=.03). On dividing the groups into no policy exposure, single policy exposure, or both exposures, the mean differences were found to be statistically significant (3.5, 7.5, and 9.4, respectively) (*P*=.03).

**Conclusions:**

In this study, a number of young physicians demonstrated a casual approach to social media activity in the context of professional medical practice. Several areas of potential educational opportunity and focus were identified: (1) online privacy, (2) maintaining digital professionalism, (3) safeguarding the protected health information of patients, and (4) the impact of existing social media policies. Prior social media instruction and/or familiarity with a social media policy are associated with an improved performance on case-based questions regarding online professionalism. This suggests a correlation between an instruction about online professionalism and more cautious online behavior.

Improving the content and delivery of social media policy may assist in preserving institutional priorities, protecting patient information, and safeguarding young professionals from online misadventure.

## Introduction

The use of social media by members of professional groups has grown substantially in the past decade. In medical practice, online activities offer potential benefits to providers, patients, and the profession [1]; and present unique challenges for users and their employers. For younger generations, unconstrained online social activity is the norm, but as these tech-savvy users transition into professional life, their attitude toward online security and professionalism may not match that of their employers or their professions. Many health care institutions embrace social media policies, although it is not clear whether these policies address misperceptions among social media users or mitigate potential pitfalls [2]. Despite the existence of social media policies, professionals continue to find themselves in ethical, professional, and/or legal trouble [3-9]. Little data are available about the perceptions and social media practices among the young medical practitioners entering the professional clinical arena. The impact of the existing social media policy on the attitude of these professionals is also unclear.

## Methods

This was a prospective observational study involving the new resident physicians in a large academic medical center. Residents were invited to participate and answer an anonymous questionnaire regarding their personal use of social media and their perceptions of appropriateness while using social media in the context of clinical medicine. Medical residents from the departments of internal medicine, family medicine, general surgery, emergency medicine, neurology, anesthesiology, neurosurgery, radiology, and obstetrics and gynecology were included. An approval was obtained from the Institutional Review Board (IRB). The 30-item questionnaire covered the following: self-reported instruction on social media use in medical school (1); self-reported familiarity with current institutional social media policy (1); case-scenario questions regarding specific tenets of the current institutional social media policy (15); understanding of social media account control options (5); prior social media account closures (2); prior unprofessional posts (2); current social media use (2); and demographics (2).

On the basis of the correct responses to the questions regarding specific tenets of the current institutional social media policy, a score was calculated for each participant (0 to a maximum of 15). Questionnaires were delivered electronically to each resident by their respective program directors during an orientation period (3-5 days before their clinical start date). In accordance with the medical center’s usual orientation procedures, each resident received printed information about the institution’s social media policy from the Graduate Medical Education (GME) office several days before starting clinical duties and receiving the invitation to attempt this questionnaire. The policy was also available for review on the institution’s Website. The study period was June 25-July 15, 2015. Upon completion of the questionnaire, the subjects were invited to review the recommended answers and explanations developed by the expert consensus. These explanations were based on the existing institutional social media policy and social media best practices. Data were analyzed using SAS 9.4 (Cary, NC), categorical variables were compared using chi-square or Fisher’s exact test as appropriate, and the correct responses to questions regarding specific tenets of the current institutional social media policy were compared between different groups using the Kruskal–Wallis analysis of variance.

## Results

Of 124 invited subjects, 70 participated in the questionnaire (response rate of 56%). Of the 70 questionnaires, 13 contained incomplete answers. Most incomplete answers were observed in the latter half of the questionnaire. The highest rate of incompletion was associated with questions 24-31 (see Multimedia Appendix 1). About 98% of the respondents were born between 1979 and 1998. The majority of respondents (60/70, 86%) then reported having a social media account (eg, Facebook, Twitter) while 51% (36/70) reported using an image messaging app (eg, Snapchat, Flickr).

The cohort of residents familiar with the current institutional policy on social media was 29% (20/70). Familiarity with the current institutional policy on social media had a statistically significant effect on response tendencies for 2 individual questionnaire items. Of those familiar with the policy who answered a question about posting a picture of a colleague, a greater percentage (63%, 12/19) selected the correct answer than those who were unfamiliar with the policy (32%, 14/44, *P*=.0204). On a question about posting an image of a patient who is “unidentifiable,” 100% of residents who reported familiarity with the social media policy answered correctly (“never okay”), compared with only 78% (32/41) of residents who were unfamiliar with the policy (*P*=.0237).

A majority of the participating residents (67%, 47/70) received formal instruction about social media use previously during medical school. Exposure to social media policies in medical school was associated with statistically significant response trends for 2 questionnaire items. When participants were asked about accepting a social network invitation from a professional acquaintance with whom no significant social or professional relationship exists, 100% of respondents with no medical school instruction about social media answered incorrectly compared with 72% (29/40) of subjects who reported prior instruction during medical school (*P*=.0125). In response to a question about interacting with patients via social media, 67% (27/40) of interns with prior medical school instruction answered correctly (“never okay”). Among interns with no prior medical school instruction on social media, only 35% (6/17) submitted a correct response regarding communication with patients via social media (*P*=.0243).

Participants were grouped according to their report of prior medical school instruction on social media and their familiarity with the current institutional social media policy. The results are summarized in Tables 1 and 2. The potential correct response total was 15; the actual range of correct responses was 0-13. Familiarity with current institutional policy was associated with an average of additional 2.2 more correct responses. Instruction on social media use during medical school similarly was associated with correct test scores for an average of 2 additional questions (see Table 1). On dividing the groups into no positive factor, either (single) positive factor, or both factors, the mean differences were found to be statistically significant (3.5, 7.5, and 9.4, respectively), using the Kruskal–Wallis test (see Table 2).

Of the participants, 39% (27/70) believed that digital information, once posted, could be permanently deleted, while 21% (15/70) were not sure. When the participants were asked, whether logging into a personal social media account while on duty in a patient care area is acceptable (not permitted by institutional policy), 27% (17/64) selected incorrect answers (“it depends” or “always”), while 5% (3/64) were “unsure.” In response to a question about posting pictures of a patient’s discrete physical finding on social media with no obvious way to identify the patient (not permitted by hospital policy), 15% (9/60) described this practice as “always ok, it depends, or unsure.” Regarding interprofessional interaction on social media, 25% (14/57) of subjects indicated that it is “always okay” to accept a social media invitation (eg, “friend request”) from a nurse with whom there is otherwise no social relationship. When the participants were asked whether it is acceptable to interact with patients on social media, which constitutes conduct that is discouraged by the social media policy, 33% (19/57) participants answered “it depends.”

**Table 1:** Average correct score stratified by institutional social media policy familiarity and medical school instruction on social media.

Parameter	*N*	Mean correct^b^	95% CI	*P*-value^a^
Familiar with institutional social media policy	20	9.0	7.6-10.4	.01
Not familiar with institutional social media policy	49	6.8	5.8-7.8	
Instruction on social media in medical school	47	8.0	7.0-9.0	.03
No instruction on social media in medical school	22	6.0	4.9-7.4	

^a^Student’s t-test, with unequal variance

^b^The possible correct score range was 0-15

**Table 2:** Added effect of medical school instruction on social media and familiarity with institutional social media policy.

Group	Number	Correct answers mean^b^	*P*-value^a^
No instruction in medical school, not familiar with policy	18	3.5	.03
Instruction on social media in medical school *only* (31), or familiar with social media policy *only* (4)	35	7.5	
Instruction on social media in medical school and familiar with social media policy	16	9.4	

^a^By Kruskal–Wallis test

^b^The possible correct score range was 0-15

## Discussion

The results of this questionnaire reveal several areas of educational opportunity and focus: (1) online privacy and preserving professional and personal identities, (2) maintaining digital professionalism, (3) safeguarding the protected health information of patients, and (4) the impact of existing social media policies and the need for improved education about social media in medicine.

### 1. Online Security and the Maintenance of Professional Persona versus Personal Identity

As 98% of the resident participants belong to the “millennial” generation, the age range of our subjects is consistent with the majority of medical professionals currently entering clinical practice. Social media is an integral part of daily life for many people in this first generation of “digital natives” and it displays very high levels of connectivity. Over 50% of the participants reported that they are either “almost always” or “mostly” online and connected. Furthermore, 80% of these digital natives “don’t worry at all” or “worry a little” about online privacy [10]. These statistics frame a scenario for trouble when highly connected, digital native users enter the medical arena, where privacy and security are professional and institutional priorities. Social media policy and best practices emphasize the importance of maintaining a distinction between personal and professional online presence.

Some of these advocate a “dual-citizen approach,” which maintains online professional and private identities by creating separate online profiles [11]. A majority of medical residents and fellows surveyed was found to have personal information on their profile page [12]. Another study of recent medical graduates revealed that the religious views, sexual orientation, and relationship status could be determined for those who did not activate the privacy settings on their profile [13]. A minority (25%) of our study participants chose “I don’t know” as the answer when asked about the effect of privacy settings on a stranger’s ability to search for them. Of the subjects considered, 53% (30/57) believed that it is “always okay” to interact with a physician colleague on social media, 49% (28/57) selected “it depends,” and 25% (14/57) selected “always okay” to accept a “friend” request from a nurse with whom there is a fleeting professional relationship and no social relationship. This type of online relationship is discouraged by the institution’s best practices guidelines. Millennial health care providers appear to have a relaxed stance toward interprofessional digital networking and may not recognize the potential ramifications of blurring their identities online. This suggests an opportunity to inform and counsel during medical school and/or during the transition into clinical practice. On addition of a new 2-hour session called “Online Social Media and Professionalism” to an existing medical school curriculum, 40% of subjects who participated in a 4-month follow-up survey reported that they had “reviewed, edited, and/or made a significant change in their online presence” [14].

Many institutions and policies discourage digital communication with patients. Communicating with patients via an open-access, unsecure site renders protected health information to be vulnerable [15]. Furthermore, interaction with patients via social media blurs the line between personal friend and health care provider. In a survey of medical residents and fellows abroad, approximately half of the respondents felt that “the doctor-patient relationship would be altered if patients discovered that their doctor had a Facebook account” [12]. Interestingly, 33% of our study subjects answered “it depends” when asked about the appropriateness of interacting with patients on social media. A vast majority (89%) of study participants agreed that providing medical advice to patients via social media is “never okay.”

### 2. Digital Professionalism and Institutional Representation

As an employee of a hospital, a physician is a representative of that facility and an ambassador of the medical profession. Furthermore, all physicians are held to local and national standards of digital conduct. For example, the Federation of State Medical Boards has policy guidelines for the appropriate use of social media in medical practice [16]. Activity in the social media world creates new, and possibly unrecognized, responsibilities. Online misadventures, intentional or inadvertent, can result in the breach of patient confidentiality, the fermenting of negative public perception, the undermining of institutional integrity, and the deterioration of professionalism. In a study of recent medical graduates, 46% of subjects had posted their pictures using alcohol and 10% showed intoxication [13]. A study of surgical residents’ Facebook posting habits found that 14% of them had posted potentially unprofessional content and 12% had posted clearly unprofessional content [7]. In this study, 49% of participants selected “it depends” while posting a picture of a social event where people are “holding alcoholic beverages.” About 26% of the participants stated that they had posted statements or photos on social media that could be considered unprofessional.

A study at a US medical school revealed differences in opinion among students about “appropriate” online content. Some students “felt nothing was inappropriate” and a minority recognized that online activity can reflect on the institution or their profession [17]. A higher incidence of clearly unprofessional Facebook content among surgical residents (12%) compared with surgical faculty (5%) has been reported. Interestingly, among the faculty, men with less than 5 years of clinical practice were associated with unprofessional online habits [6,7]. More conservative attitudes about the appropriateness of Facebook posts have been described among faculty, women, and older participants [18], further emphasizing generational differences in attitudes and habits toward online professionalism. This suggests that younger users see online accountability from a perspective of personal risk only. This validates the need for instruction about the broader impacts of online behavior.

### 3. Patient Confidentiality

Patient confidentiality achieves wide consensus in the social media discussion. Protected health information and patient privacy should never be compromised by social media or any other form of communication. However, some examples of confidentiality breaches in medicine involve an unwitting rule breaker whose harmful actions are unintentional. For example, a physician was fined by the state medical board and fired by her hospital after posting a detailed account of a patient encounter [3]. Other events described in the media suggest poorer judgment and malicious intent, for example, providers have posted unflattering or personal information about patients on social media sites [5] and have posted inappropriate pictures on image-messaging accounts [4]. Whether intentional or unintentional, the potential for compromising patient information on social media is real and the stakes are quite high.

In this study, subjects were asked about the appropriateness of posting a comment (including a description of gender, approximate age, and injury pattern) about a patient encounter immediately after a mass casualty event. A surprising 25% of respondents selected “always,” “it depends,” or “not sure.” Rarity of disease or injury and proximity in timing or geographic location make identification of the patient in this scenario feasible. Therefore, posting such information could result in the unintentional release of protected health information and, as such, is forbidden at the study institution. This reveals an opportunity to alert our physicians about the hazards to patient privacy in posting seemingly anonymous or harmless information.

### 4. Social Media Policy and Education

Our results suggest a positive effect from social media instruction and policy familiarity on participants’ answer selection in the survey. Although the impact upon online *conduct* of these participants is unknown, improved knowledge and awareness on this topic is desirable. Conventional approaches to professionalism taught in a well-established educational framework may fall short when applied to novel online scenarios in professional medicine, for example, despite reported medical school instruction, 32.5% of interns in this study group answered incorrectly about interacting with patients online. Is this the result of ineffective education or poor knowledge retention? The authors surmise that it is a result of challenges with real-world application at the user level. Traditional professionalism required during observable human interactions is easily recognized by the learner and has a finite duration. In contrast, social media has the power to project personal activities and musings into the public sphere to lay consumption with infinite duration, even if the user has no such intention. A new approach is needed to address the phenomenon of professionalism in the digital age, or “e-professionalism” [19]. Instruction on “e-professionalism” must provide contextual relevance for the learner and instill an appreciation for the potential reach and impact of online activity.

### Bedside Distraction

Another area of digital professionalism not yet adequately studied is the impact of digital networking on clinical performance in the real world. The ubiquity and accessibility of mobile connectivity allows online communication to thrust its way into the clinical environment and, at times, to the bedside. As such, digital professionalism necessarily applies to the clinical workspace. When participants were asked whether logging onto a personal social media account while on duty in a patient care area was acceptable, 27% (17/64) selected “it depends,” while 5% (3/64) were “unsure.” The social media policy at the study site prohibits this type of activity. When participants were asked whether receiving message notifications on a mobile during work hours in the hospital constitutes a patient safety risk, 9% (5/57)of subjects characterized this practice as “no risk,” while 70% (40/57) selected “possible risk.” Most would probably contend that receiving audible notifications during patient care activities constitutes, at minimum, a distraction. The presence of distractions and the impact of interruptions on medical care are well documented [20-24]. A case commentary of online messaging distraction resulting in significant medical error has been reported [25]. The impact of messaging alerts and social media activity on physician focus, task performance, and patient safety is an area that requires further investigation.

### Looking Ahead

While establishing social media policies, medical institutions must balance several variables; the needs of their professional employees, the potential for social media to improve health care, and their legal and ethical obligations to the patients and communities they serve [26]. Given the high stakes nature of this new digital phenomenon, education has been geared toward establishing acceptable behavioral parameters and has been rooted in risk prevention [2,14,15]. Some have raised concerns that an overemphasis on control, security, and risk avoidance may impede the potential benefits that social media can provide [27].

A sophisticated understanding and awareness of the potential benefits and hazards of social media are a prerequisite to responsible online activity. Efforts to raise awareness among medical students and physicians in transition may help shape desired online behaviors. Evidence supporting this approach includes a report of a “raised perception of risk” among medical students after hearing personal stories, warnings by school officials, and media reports [17]. While the debate about effective and comprehensive instruction for online users continues, there are resources available that provide current and relevant information about judicious use of social media in the context of medical practice [15,28,29].

### Limitations

Some subjects were previously enrolled as students at the study institution so their “formal instruction” on social media usage may have contributed to familiarity with the current institutional policy. We did not identify those individual subjects. The study is limited by a relatively small sample size (*n*=124 invited subjects) at a single academic medical institution. Questionnaires were delivered electronically via their respective program director during a very busy onboarding process, which may contribute to a response bias. Subjects may have felt compelled to complete the survey or may not have given proper attention to each question, given all the activity that goes on during the onboarding process. Only incoming house officers were surveyed which may make some of the conclusions less generalizable to all house officers.

### Conclusion

Young physicians demonstrate a casual approach to social media activity in the context of professional medical practice. However, social media instruction and/or familiarity with the social media policy are associated with more cautious perceptions about online behavior. Furthermore, assessment of perceptions and practices of new employees in a health care environment may help improve the content and delivery of policy information. This approach may help to preserve institutional priorities, protect patient information, and safeguard young professionals from online misadventures.

## Appendix

Multimedia Appendix 1
