# Supplementary material for: Social Media in Professional Medicine: New Resident Perceptions and Practices
Source: J Med Internet Res. 2016 Jun 9;18(6):e119. doi: 10.2196/jmir.5612 (PMC4919547; doi:10.2196/jmir.5612)
Supplement: Multimedia Appendix 1 [file jmir_v18i6e119_app1.pdf]

Appendix A

Questionnaire on the Use of Social Media by Medical Professionals

1. Do you currently have a social media account (Facebook, Twitter, etc) for your own private use? Y/N
2. Do you currently use an image messaging application (Snapchat, Instagram, etc) for your own private use? Y/N
3. Did you receive formal instruction about the use of social media during medical school? Y/N
4. Are you familiar with the social media policy at this institution? Y/N
5. Have you ever closed a personal social media account for personal reasons? Y/N
6. Have you ever closed a personal social media account for professional reasons? Y/N
7. If you have a personal social media account, do you know the current privacy setting of your account? Y/N
8. Can you prevent a person/patient from searching for your social media profile? Y/N
9. Can *anyone* search for you on your social media account regardless of privacy setting? Y/N
10. Is it possible to change settings to control who can tag you on a photo or image? Y/N
11. I can permanently delete a post or other material I have posted to my social media account. Y/N
12. I have posted statements and/or photos on social media that could be considered unprofessional. Y/N
13. I have posted statements and/or photos on social media that I now regret because they are unprofessional. Y/N
14. While on duty in the hospital, you are in a patient care area. Your patient(s) is stable, and does not require intervention. You are immediately available should your patient require assistance of any kind. Is it professionally acceptable to access your social media account? Never/ It depends / Always / Not sure
15. Is it okay to post a picture of a residency event in a conference room, as long as everyone consents to having their picture posted? Never/It depends/Always/Not sure
16. Is it okay to post a picture of an off-campus, non-sanctioned event (e.g., baseball game) in which some of your resident colleagues are present? Never/It depends/Always/Not sure
17. You attend a departmental BBQ and your colleagues pose for a group photo. They all agree to have their picture posted on-line. You post this picture on your social media page to help promote the program. Never okay/It depends/Always okay/not sure
18. You participate in a “night-out” with friends. You snap a picture of a group in which some people are holding alcoholic beverages. You want to post this to your social media account and determine this is: Never okay/It depends/Always okay/Not sure.
19. Your patient has a very interesting rash on their back. You take a picture of this rash for the chart. There is no way to identify the patient from the picture. You think it would be highly educational to share this finding with your colleagues. Is it okay to post the picture to your social media account? Never/ It depends/ Always / Not sure
20. You are reviewing an interesting CT of a patient. There is no identifying information about the patient on this diagnostic image. You decide to post the image on social media. Never okay /It depends/ Always okay/Not sure
21. You are called to the ED for a mass casualty event. The department is crowded and you are proud to be a part of a coordinated effort to help the community. You take a picture and a short video on your mobile device of the entire scene. Providers and patients are visible in the frame but it is impossible to make out individual faces. You consider posting this on your social media account. You decide this is: Never okay/ It depends/ Always okay/Not sure
22. You take your patient from the mass casualty event to the OR for a severe penetrating injury. After successfully participating in the care of this patient, you post the following statement to your social media

account, “Wow –crazy day! Took a young boy to the OR for nasty impalement. He’s doing great. I love my job.” This is: Never okay/It depends/Always okay/Not sure

23. You and a resident colleague take a picture of yourselves in front of a patient tracking board. There are no patients listed on the board, and there are no patients in the background. Is this picture ok to post? Never okay/ It depends/Always okay/Not sure

24. Is it okay to interact with physician colleagues on social media as an acquaintance (e.g., “Facebook friend”)? Never okay /It depends/ Always okay/Not sure
25. You have set your social media account and/or messaging application to provide notifications on your mobile device. Receiving notifications on your mobile device while on duty in the hospital constitutes a: Definite Patient Safety Risk/Possible Patient Safety Risk/No Patient Safety Risk
26. You receive a “friend” request on Facebook (or similar request on a different social media platform) from a nurse with whom you have worked briefly but with whom you otherwise have no social relationship. Is it okay to accept this “friend” request? Never okay /It Depends/ Always okay/Not sure
27. You receive a “friend” request on Facebook from a radiology technician who is a “friend of a friend” but with whom you have neither worked nor met. Is it okay to accept this “friend” request? Never/It Depends/ Always/Not sure
28. Is it okay to interact with patients on social media? Never/It depends/Always/Not sure
29. Is it okay to provide medical advice to patients via social media? Never okay/It depends/always okay/Not sure
30. Are you: Female   Male
31. Please Indicate your age: 18-25   26-35   35-45   >46
